# ST2 and REG3α as Predictive Biomarkers After Haploidentical Stem Cell Transplantation Using Post-transplantation High-Dose Cyclophosphamide

**DOI:** 10.3389/fimmu.2019.02338

**Published:** 2019-10-09

**Authors:** Laura Solán, Mi Kwon, Diego Carbonell, Nieves Dorado, Pascual Balsalobre, David Serrano, María Chicano-Lavilla, Javier Anguita, Jorge Gayoso, José Luis Díez-Martín, Carolina Martínez-Laperche, Ismael Buño

**Affiliations:** ^1^Department of Hematology, Gregorio Marañón General University Hospital, Madrid, Spain; ^2^Gregorio Marañón Health Research Institute (IiSGM), Madrid, Spain; ^3^Department of Medicine, Complutense University of Madrid, Madrid, Spain; ^4^Genomics Unit, Gregorio Marañón Health Research Institute (IiSGM), Gregorio Marañón General University Hospital, Madrid, Spain

**Keywords:** hematopoietic cell transplantation, haploidentical, non-relapse mortality, graft vs. host disease, biomarkers, ST2, REG3α

## Abstract

Allogenic hematopoietic stem cell transplantation (allo-HSCT) is a curative procedure for several hematological malignancies. Haploidentical HSCT (haplo-HSCT) using high-dose post-transplantation cyclophosphamide (PTCy) makes transplantation possible for patients with no HLA-matched sibling donor. However, this treatment can cause complications, mainly infection, graft-vs.-host disease (GVHD), and conditioning-related toxicity. In recent years, different biomarkers in the form of tissue-specific proteins have been investigated; these may help us to predict complications of allo-HSCT. In this study we explored two such biomarkers, suppression of tumorigenicity 2 (ST2) and regenerating islet-derived 3α (REG3α), in the largest series reported of T cell–replete haplo-HSCT with PTCy. Plasma samples drawn from 87 patients at days +15 and +30 were analyzed. ST2 and REG3α levels at day +15 were not associated with post-transplant complications. ST2 levels at day +30 were higher in patients with grade II-IV acute GVHD, mainly those who received reduced intensity conditioning (RIC; median 2,503 vs. 1,830 ng/ml; *p* = 0.04). Of note, patients with higher plasma ST2 levels at day +30 also presented a higher incidence of non-relapse mortality (HR, 7.9; *p* = 0.004) and lower 2-year overall survival (25 vs. 44 months; *p* = 0.02) than patients with lower levels. Patients with REG3α levels higher than 1,989 pg/ml at day +30 presented a higher incidence of acute gastrointestinal GVHD in the whole cohort (HR, 8.37; *p* = 0.003) and in the RIC cohort (HR 6.59; *p* = 0.01). These data suggest that measurement of ST2 and REG3α might be useful for the prognosis and prediction of complications in patients undergoing haplo-HSCT with PTCy.

## Introduction

Post-transplant high-dose cyclophosphamide (PTCy) provides effective prophylaxis against graft-vs.-host disease (GVHD) in patients undergoing unmanipulated haploidentical stem cell transplantation (haplo-HSCT). It has enabled extended use of haploidentical donors for treatment of hematologic malignances with unmanipulated peripheral blood stem cells (PBSC) (1–3). Several reports have shown comparable outcomes between haplo-HSCT and historical series of matched related donors, matched unrelated donors and mismatched unrelated donors (4–8).

Despite these clinical successes, the cumulative incidence of non-relapse mortality (NRM) at 2 years in haplo-HSCT is up to 25%, with the most important complications being GVHD and infections (9, 10). Pre-transplant clinical scales can help to identify patients with a higher risk of mortality during the transplant process (11–13), although these are imprecise and non-specific.

Similarly, the prediction and diagnosis of acute GVHD (aGVHD) is often difficult and requires clinical data to be combined with histopathological confirmation, an approach that is not always possible. The severity of symptoms at onset of GVHD does not accurately define risk, and all patients are treated similarly with high-dose systemic corticosteroids as initial therapy (14). Thus, in the last few years, various biomarkers have been studied to enable the prediction, diagnosis, and prognosis of NRM and GVHD (15–17). Such biomarkers could potentially guide treatment decisions, leading to intensive clinical surveillance of patients at high risk of developing complications. Plasma biomarkers have been identified and validated as promising diagnostic and prognostic tools for post-transplant complications. These biomarkers can facilitate timely and selective therapy but should be more widely validated and incorporated into a new grading system for stratification of risk and better-customized treatment (18, 19). Two of the most widely studied biomarkers in HLA-identical allo-HSCT are regenerating islet-derived 3 alpha (REG3α) and suppression of tumorigenicity 2 (ST2). REG3α is produced in the pancreas and small intestine, and its expression is enhanced during inflammatory processes. It has been postulated that REG3α levels are directly proportional to the endothelial damage caused by GVHD. This biomarker is useful in the diagnosis of gastrointestinal aGVHD, since it correlates with inflammatory activity in GVHD and can distinguish between other causes of diarrhea (e.g., autoimmune disease, toxicity, and infection). An estimate of total damage to the mucosal barrier may also help to explain the prognostic value of REG3α with respect to response to therapy and NRM (20–22). ST2, on the other hand, is a member of the interleukin-1 receptor family and has been directly related to the risk of treatment-resistant aGVHD and 6-month NRM after onset of aGVHD independently of clinical severity (23–25). Most of these studies are performed in identical HLA or umbilical cord–based allo-HSCT. To our knowledge, only one study has been performed on patients receiving PTCy. Kanakry et al. (15) explored seven plasma-derived proteins in 58 HLA-haploidentical and 100 HLA-matched related or unrelated T cell–replete bone marrow transplants. Levels of ST2 and REG3α at day +30 predicted occurrence of NRM at 3 months in both cohorts. In this context, our objective was to analyze plasma levels of REG3α and ST2 at days +15 and +30 after transplant and to correlate them with complications in a large cohort of patients who underwent unmanipulated haplo-HSCT with high-dose PTCy.

## Patients and Methods

### Patient Population

We retrospectively analyzed plasma samples from 110 consecutive patients who underwent haplo-HSCT between 2009 and 2016 at a single center. We excluded 23 cases, nine due to early death (before day +30) and 14 due to lack of plasma samples. Only one patient had pre-transplant anti-HLA antibodies which, after treatment according to the center protocol, were undetectable at the day of infusion. All patients received PTCy 50 mg/kg/day (days +3, +4), mycophenolate mofetil, and cyclosporine as GVHD prophylaxis from day +5. Donor lymphocyte infusion was performed in 10 patients, and a further 3 patients received CD34+-selected stem cell boosts.

### Sample Collection and Processing

Samples were collected at days +15 and +30 after transplantation. All patients analyzed had at least one sample from one of the two timepoints. Plasma was obtained from peripheral blood samples by refrigerated (4°C) centrifugation at 2,000 rpm for 30 min in the 2–6 h following extraction. Samples were aliquoted without additives into cryovials and stored immediately at −80°C. ST2 and REG3α were detected using ELISA according to the manufacturer's instructions (Critical Diagnostics, San Diego, California, USA for ST2 and MBL International Corp, Woburn, Massachusetts, USA for REG3α). For the determination of ST2 and REG3α, samples (diluted 1:10 in the case of ST2) and standards were run in duplicate, and absorbance was measured using the VICTOR2 D fluorometer™ (multilabel plate reader). ST2 plasma levels were available on day +15 in 70 patients and on day +30 in 66 patients. Similarly, REG3α plasma levels samples were available on day +15 and +30 in 75 and 71 patients, respectively.

### Statistical Analysis

Numerical and categorical variables were expressed as median (range) and frequency (percentage), respectively. The Mann-Whitney test was used to compare differences between two independent variables. The determination of the best cut-off for ST2 and REG3α levels to stratify patients was derived from receiver operating characteristic (ROC) curves. Also, time-dependent ROC curves for competing endpoints were calculated. Predictive accuracy was estimated based on the area under the ROC curve at 3, 6, 9, and 12 months for NRM, at 500, 1,000, 1,500, and 2,000 days for death and at 30, 60, 90, and 180 days for aGVHD. Univariate regression analysis was performed using Cox regression [hazard ratio (HR)] and the Fine-Gray model was performed to assess the association of each biomarker with postransplant outcomes. Estimates of grade III–IV aGVHD and relapse were calculated using cumulative incidence rates. For the analysis of GVHD, those patients who received donor lymphocyte infusion, relapsed, or died before day 180 were censored. Overall survival (OS) and event-free survival (EFS) were calculated using the Kaplan-Meier method. Survival curves were compared using the log-rank test. Statistical analyses were performed using SPSS v18 for Windows (SPSS Inc., Chicago, Illinois, USA). Cumulative incidence rates were calculated using the statistical package R ver. 3.3.2 (https://cran.r-project.org).

## Results

Data from 87 patients who underwent haplo-HSCT with PTCy (Table 1) were retrospectively analyzed. The median follow-up period was 41 months (range, 15–109 months). Median age was 46 years (range, 16–66), and the most common indications for transplantation were acute myeloid leukemia (32%) and Hodgkin's lymphoma (23%). Peripheral blood was the main stem cell source used. The cumulative incidence of grade II–IV and grade III–IV aGVHD at 100 days was 51.5 and 14.2%, respectively. The cumulative incidence of moderate-to-severe chronic GVHD at 2 years was 10%, and that of relapse and NRM at 2 years was 27 and 22.2%, respectively. Two-year OS and EFS were 62 and 50%, respectively.

**Table 1:** Clinical features of patients and transplants.

**Characteristics**	**Value**
Recipient median age, years (range)	46 (16–66)
Recipient sex, female/male, *n*	25/62
Female donor/Male recipient, *n* (%)	28 (32)
Donor median age, years (range)	40 (14–68)
Primary malignancy, *n* (%)	
Acute myeloid leukemia	28 (32)
Hodgkin lymphoma	20 (23)
Non-Hodgkin lymphoma	11 (13)
Acute lymphoblastic leukemia	9 (10)
Myelodysplastic syndrome	7 (8)
Myelofibrosis	3 (3)
Multiple myeloma	2 (2)
Chronic lymphocytic leukemia	2 (2)
Aplasia	1 (1)
Others	4 (5)
Disease risk index, *n* (%)	
Very high + high	35 (40)
Intermediate	50 (57)
Low	2 (2)
Pretransplant disease status, *n* (%)	
Complete remission	46 (53)
Partial remission	33 (38)
Active disease	8 (10)
Previous autologous transplant, *n* (%)	28 (32)
Previous allogeneic transplant, *n* (%)	10 (11)
Recipient/donor CMV serostatus, *n* (%)	
Matched	58 (67)
Mismatched	26 (30)
Missing	2 (2)
Conditioning regimen intensity, *n* (%)	
Myeloablative^*^	35 (40)
Reduced intensity conditioning ^∧^	52 (60)
Stem cell source, *n* (%)	
Bone marrow	10 (12)
Peripheral blood	77 (88)
CD34+ cell dose infused, median (range)	
Bone marrow	3.07 × 10^6^/kg (1.07–4.73)
Peripheral blood	5.34 × 10^6^/kg (2.24–11.4)

* Myeloablative conditioning regimen: Fludarabine 40 mg/m^2^ for 4 days and Busulfan 3.2 mg/kg 3 or 4 days.

∧ Reduced intensity conditioning regimen: Fludarabine 30 mg/m^2^ for 4 days, Cyclophosphamide 14.5 mg/kg on days −6 and −5 and Busulfan 3.2 mg/kg from day-3 for 1 or 2 days.

### ST2

No association was found between median ST2 levels and clinical variables (age, sex, stem cell source, donor sex, hematological malignancy, disease status at transplant, hematopoietic cell transplantation–associated comorbidity, previous transplant, conditioning regimen intensity, and number of infused CD34+ cells; data not shown). We correlated median ST2 levels with post-transplant complications for the whole cohort (Table 2) and for patients who received only reduced intensity conditioning (RIC; Table 3). No differences were found between the occurrence of post-transplant outcomes and ST2 levels in patients who received myeloablative conditioning (data not shown).

**Table 2:** Association between ST2 levels at day +15 and +30 and GVHD (acute and chronic), NRM, relapse, and OS in the whole cohort (*n* = 87).

**Whole cohort (*****n*** **=** **87)**		**ST2 +15 (ng/ml)**	***p*-value**	**ST2 +30 (ng/ml)**	***p*-value**
		**Median (range)**		**Median (range)**	
aGVHD II-IV	Yes	2,296 (376–4,903)	0.85	2,466 (524–5,275)	0.37
		*n* = 38		*n* = 38	
	No	2,319 (1,045–4,633)		2,127 (1,048–3,981)	
		*n* = 29		*n* = 32	
aGVHD III–IV	Yes	2,067 (376–3,933)	0.17	2,499 (524–5,275)	0.28
		*n* = 12		*n* = 10	
	No	2,337 (688–4,903)		2,154 (809–4,572)	
		*n* = 55		*n* = 60	
Chronic GVHD (moderate and severe)	Yes	1,991 (860–4,320)	0.67	2,019 (524–5,100)	0.92
		*n* = 11		*n* = 13	
	No	2,319 (376–4,903)		2,163 (809–5,275)	
		*n* = 47		*n* = 49	
Relapse	Yes	2,319 (1045–4,903)	0.61	2,317 (524–6,072)	0.35
		*n* = 19		*n* = 22	
	No	2,311 (376–4,633)		2,146 (809–6,072)	
		*n* = 51		*n* = 53	
Non-relapse mortality	Yes	2,315 (376–4,633)	0.68	2,975 (1,352–6,072)	0.02^*^
		*n* = 15		*n* = 16	
	No	2,260 (688–4,311)		2,015 (809–5,275)	
		*n* = 37		*n* = 38	
Status at las follow up	Dead	2,315 (376–4,903)	0.53	2,499 (524–6,072)	0.08
		*n* = 29		*n* = 34	
	Alive	2,319 (688–4,311)		2,015 (869–5,275)	
		*n* = 41		*n* = 41	

* *Indicates statistical significance*.

**Table 3:** Association between ST2 levels at day +15 and +30 and GVHD (acute and chronic), NRM, relapse, and OS in the RIC cohort (*n* = 52).

**RIC cohort (*****n*** **=** **52)**		**ST2 +15 (ng/ml)**	***p*-value**	**ST2 +30 (ng/ml)**	***p*-value**
		**Median (range)**		**Median (range)**	
aGVHD II-IV	Yes	2,287 (376–4,903)	0.89	2,503 (524–5,275)	0.045^*^
		*n* = 24		*n* = 26	
	No	2,319 (1,045–3,569)		1,830 (1,394–3,529)	
		*n* = 15		*n* = 17	
aGVHD III-IV	Yes	2,067 (376–3,109)	0.27	2,496 (524–5,275)	0.37
		*n* = 8		*n* = 7	
	No	2,319 (688–4,903)		2,019 (809–4,572)	
		*n* = 31		*n* = 36	
Chronic GVHD (moderate and severe)	Yes	1,809 (869–2,333)	0.09	1,868 (524–5,100)	0.87
		*n* = 5		*n* = 9	
	No	2,319 (376–4,903)		2,146 (809–5,275)	
		*n* = 29		*n* = 29	
Relapse	Yes	2,319 (1,045–4,903)	0.59	2,527 (524–5,715)	0.79
		*n* = 11		*n* = 13	
	No	2,282 (376–4,472)		2,085 (809–6,072)	
		*n* = 31		*n* = 35	
Non-relapse mortality	Yes	2,282 (376–4,472)	0.95	3,299 (1,820–6,072)	0.004^*^
		*n* = 11		*n* = 13	
	No	2,267 (688–4,150)		1,830 (809–5,275)	
		*n* = 20		*n* = 22	
Status at las follow up	Dead	2,293 (376–4,903)	0.68	2,709 (524–6,072)	0.048^*^
		*n* = 19		*n* = 23	
	Alive	2,319 (688–4,150)		1,935 (809–5,275)	
		*n* = 23		*n* = 25	

* *Indicates statistical significance*.

In both groups (whole and RIC cohort), we observed that ST2 levels at day +15 were not associated with post-transplant complications (Tables 2, 3). Instead, at day +30, median ST2 levels were higher in patients with grade II-IV aGVHD, mainly in those who had received RIC (2,503 vs. 1,830 ng/ml, *p* = 0.045). Of note, patients with higher ST2 plasma levels at day +30 had a higher incidence of NRM and lower OS than those with lower levels in both, the whole cohort [2,975 vs. 2,015 ng/ml (*p* = 0.02); 2,499 vs. 2,015 (*p* = 0.08), respectively] and the RIC cohort: (3,299 vs. 1,830 ng/ml, *p* = 0.004; 2,709 vs. 1,935 ng/ml, *p* = 0.045, respectively; Tables 2, 3).

**Figure 1:**
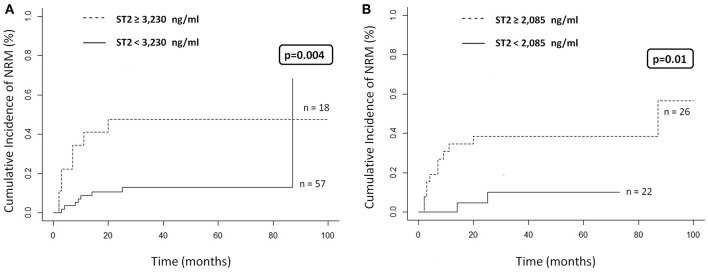
**(A)** Cumulative incidence of NRM according to ST2 levels at day +30 (cut-off 3,230 ng/ml) in the whole cohort (*n* = 75). **(B)** Cumulative incidence of NRM according to ST2 levels at day +30 (cut-off 2,085 ng/ml) in RIC patients (*n* = 48).

Based on these results, we calculated the best cut-off for ST2 levels at day +30 according to aGVHD, NRM, and OS derived from ROC curves. We were unable to find an optimal cut-off ST2 level to stratify patients correctly regarding development of aGVHD in either cohort (data not shown). Univariate analysis was performed in the whole cohort and in the RIC cohort and included a comparison between clinical variables and ST2 levels and incidence of NRM and death. We found that only ST2 levels at day +30 (HR, 7.9; *p* = 0.004) were associated with the occurrence of NRM (Table 4, Figure 1A). Similar results were obtained in the RIC cohort; in the univariate analysis, only ST2 levels at day +30 (HR, 5.4; *p* = 0.01) correlated with a higher incidence of NRM (Table 4, Figure 1B). In both cohorts, most patients died of infection or GVHD (Table 5). However, in the whole cohort, five patients presented NRM (cataloged as GVHD) with ST2 levels <3,230 ng/ml; four of these five patients died of a lower respiratory tract infection during immunosuppressive treatment for GVHD. The remaining patient died of febrile syndrome (no microbiological isolates) associated with polyserositis and elevation of liver enzymes compatible with GVHD (no confirmatory biopsy).

**Table 4:** Association between ST2 levels at day +30, clinical variables and cumulative incidence of NRM and death in the whole cohort (*n* = 87) and the RIC cohort (*n* = 52).

**Variables**	**Whole cohort**				**RIC cohort**			
	**NRM**		**Death**		**NRM**		**Death**	
	**HR**	***p*-value**	**HR**	***p*-value**	**HR**	***p*-value**	**HR**	***p*-value**
Age, >50 years	0.15	0.6	0.1	0.7	0.08	0.7	0.37	0.5
Female sex	0.16	0.6	0.1	0.6	0.55	0.4	0.24	0.6
Sorror >3	0.16	0.6	0.3	0.5	0.12	0.7	0.24	0.6
Previous HSCT	0.01	0.8	0.7	0.3	0.07	0.7	0.38	0.5
Underlying disease not AML	2.1	0.2	0.08	0.7	0.5	0.4	0.02	0.8
Stem cell source (BM)	1.2	0.2	0.6	0.4	2.3	0.1	0.79	0.3
Infused TNC >6^*^10^8^/kg	0.18	0.6	0.005	0.9	0.07	0.7	0.007	0.9
RIC conditioning regimen	2.2	0.13	0.07	0.7				
ST2 (per 1,000 units)	7.9	0.004^*^	–	–	5.43	0.01^*^	–	–
ST2 (per 1,000 units)	–	–	5.49	0.01^*^	–	–	3.47	0.05^*^

* *Indicates statistical significance*.

**Table 5:** Non-relapse mortality.

**Cause, *n* (%)**	**NRM (Whole cohort)**		**NRM (RIC cohort)**	
	**ST2 >3,230 ng/ml (*n* = 8)**	**ST2 <3,230 ng/ml (*n* = 8)**	**ST2 >2,085 ng/ml (*n* = 11)**	**ST2 <2,085 ng/ml (*n* = 2)**
aGVHD	1 (13)	4 (50)	3 (27)	1 (50)
cGVHD	1 (13)	1 (13)	1 (10)	1 (50)
Infection	3 (37)	1 (13)	3 (27)	0
Non-bacterial endocarditis	1 (13)	0	1 (10)	0
Secondary neoplasms	2 (25)	0	2 (17)	0
Ischemic heart disease	0	2 (25)	1 (10)	0

Likewise, we found that only plasma ST2 levels >1,882 ng/ml on day +30 were associated with death (including relapse of the underlying disease) both in the whole cohort (HR, 5.49; *p* = 0.01) and in the RIC cohort (HR, 3.47; *p* = 0.05) (Table 4).

**Figure 2:**
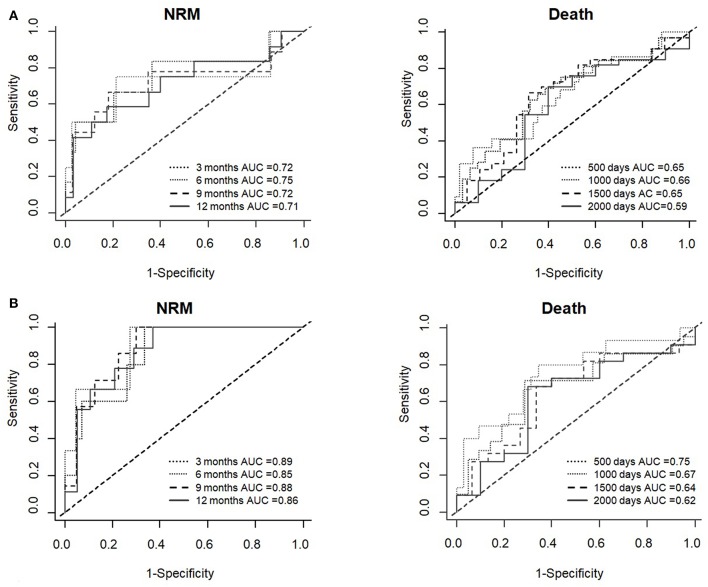
Time-dependent ROC curves for NRM and death using ST2 levels at day +30 in both, the whole cohort **(A)** and the RIC cohort **(B)**.

To confirm the association of ST2 with NRM and death, the linear effect of the biomarker on the CI was estimated using the Fine-Gray model in both cohorts (Table 6). ST2 levels at day +30 were significantly associated with greater CI of NRM and death (SHR 1.7; *p* = 0.007 and SHR 1.5; *p* = 0.01, respectively, in the whole cohort and SHR 1.9; *p* = 0.001 and SHR 1.6; *p* = 0.01 in the RIC cohort). Moreover, time-dependent ROC curves, generated to assess the overall accuracy of ST2 for predicting outcomes, showed high area under the curve (AUC) values for both NRM and death (respectively, 0.72 at 3 months and 0.65 at 500 days in the whole cohort, as well as 0.89 at 3 months and 0.75 at 500 days in the RIC cohort; Figure 2).

**Table 6:** Univariable associations between ST2 levels at day +30 and clinical variables with NRM and death in the whole and RIC cohorts using the Fine-Gray model.

**Variables**	**Whole cohort**				**RIC cohort**			
	**NRM**		**Death**		**NRM**		**Death**	
	**SHR (95%CI)**	***p*-value**	**SHR (95%CI)**	***p*-value**	**SHR (95%CI)**	***p*-value**	**SHR (95%CI)**	***p*-value**
Age, >50 years	1.2 (0.4–2.7)	0.7	1.1 (0.6–2.1)	0.8	1.1 (0.4–2.9)	0.9	1.2 (0.6–2.7)	0.6
Female sex	0.8 (0.3–2.1)	0.6	1.2 (0.6–2.4)	0.6	0.6 (0.2–1.9)	0.4	1.2 (0.5–2.8)	0.7
Sorror >3	0.2 (0.5–2.7)	0.7	1.1 (0.6–2.1)	0.7	1.04 (0.4–2.8)	0.9	1.1 (0.5–2.4)	0.9
Previous HSCT	0.8 (0.2–4.1)	0.8	1.5 (0.7–3.5)	0.3	0.8 (0.2–3.8)	0.7	1.3 (0.5–3.4)	0.6
Underlying disease not AML	2.1 (0.8–5.2)	0.1	1.2 (0.7–2.3)	0.5	1.6 (0.5–5.1)	0.4	1.1 (0.5–2.6)	0.8
Stem cell source (BM)	0.5 (0.2–1.7)	0.3	0.8 (0.3–2.1)	0.6	0.4 (0.1–1.3)	0.1	0.7 (0.2–1.9)	0.5
Infused TNC >6 × 10^8^/kg	1.2 (0.5–2.9)	0.6	0.9 (0.5–1.8)	0.7	1.1 (0.4–2.9)	0.9	0.9 (0.4–2.2)	0.9
RIC conditioning regimen	2.6 (0.9–8)	0.08	1.3 (0.7–2.5)	0.4				
ST2 (per 1,000 units)	1.7 (1.2–2.6)	0.007^*^	–	–	1.9 (1.3–2.8)	0.001^*^	–	–
ST2 (per 1,000 units)	–	–	1.5 (1.1–2.1)	0.01^*^	–	–	1.6 (1.1–2.1)	0.01^*^

* *Indicates statistical significance*.

**Figure 3:**
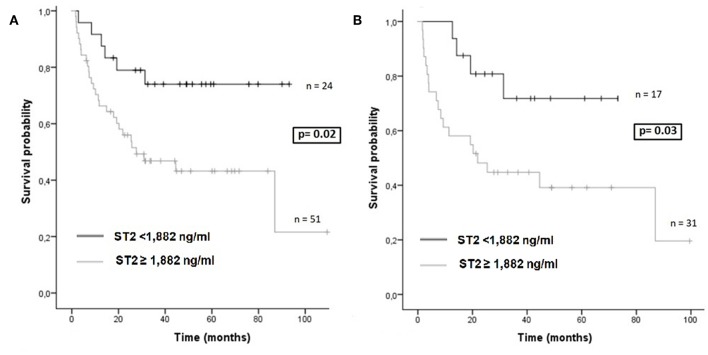
**(A)** Overall survival according to ST2 levels at day +30 (cut-off 1,882 ng/ml) in the whole cohort (*n* = 75). **(B)** Overall survival according to ST2 levels at day +30 (cut-off 1,882 ng/ml) in RIC patients (*n* = 48).

Mean OS at 2 years was higher in patients with low ST2 levels at day +30 in the whole cohort (44 vs. 25 months, *p* = 0.02, Figure 3A) and in the RIC cohort (not reached vs. 21 months, *p* = 0.03, Figure 3B).

In our study, we did not find differences between ST2 levels at days +15 and +30 and therapy-resistant GVHD. This may be due to the low number of patients (five) who showed resistance to treatment with steroids.

### REG3α

Plasma concentrations of REG3α at day +15 were too low in all samples, which prevented us from including the results obtained. We analyzed the association between REG3α levels at day +30 and the usual clinical variables (age, sex, stem cell source, donor sex, hematological malignancy, disease status at transplant, conditioning regimen intensity, and number of infused CD34+ cells) and found no association (data not shown). We also analyzed the association between REG3a levels and post-transplant complications, namely, GVHD (acute and chronic), NRM, relapse, and OS (Table 7).

**Table 7:** Association between REG3α levels at day +15 and +30 and GVHD (acute and chronic), NRM, relapse, and status.

**Whole cohort (*****n*** **=** **87)**		**REG3α +15 (pg/ml)**	***p*-value**	**REG3α +30 (pg/ml)**	***p*-value**
		**Median (range)**		**Median (range)**	
aGVHD II–IV	Yes	128 (0–2,983)	0.17	1,358 (0–7,798)	0.09
		*n* = 35		*n* = 37	
	No	14 (0–2,311)		500 (0–7,491)	
		*n* = 28		*n* = 30	
aGVHD III–IV	Yes	189 (0–2,983)	0.31	1,068 (0–7,798)	0.92
		*n* = 10		*n* = 10	
	No	110 (0–2,311)		1,055 (0–7,491)	
		*n* = 53		*n* = 57	
GI aGVHD	Yes	59 (0–2,475)	0.55	2,483 (0–5,904)	0.19
		*n* = 11		*n* = 10	
	No	134 (0–2,983)		1,011 (0–7,798)	
		*n* = 52		*n* = 57	
Chronic GVHD (moderate and severe)	Yes	0 (0–1,008)	0.08	895 (0–5,904)	0.97
		*n* = 11		*n* = 13	
	No	145 (0–2,983)		1,106 (0–7,798)	
		*n* = 44		*n* = 47	
Relapse	Yes	34 (0–2,311)	0.40	1,183 (0–7,491)	0.65
		*n* = 19		*n* = 19	
	No	146 (0–2,983)		1,042 (0–7,798)	
		*n* = 47		*n* = 52	
Non-relapse mortality	Yes	355 (0–1,928)	0.39	1,161 (0–5,904)	0.10
		*n* = 14		*n* = 15	
	No	119 (0–2,983)		500 (0–7,798)	
		*n* = 34		*n* = 38	
Status at las follow up	Dead	137 (0–1,928)	0.92	1,183 (0–7,491)	0.08
		*n* = 28		*n* = 31	
	Alive	119 (0–2,983)		702 (0–7,798)	
		*n* = 38		*n* = 40	

We did not find any association between levels of REG3α at day +30 and chronic GVHD or relapse. Similarly, no differences were found when we carried out these analyses in the RIC cohort (data not shown).

**Figure 4:**
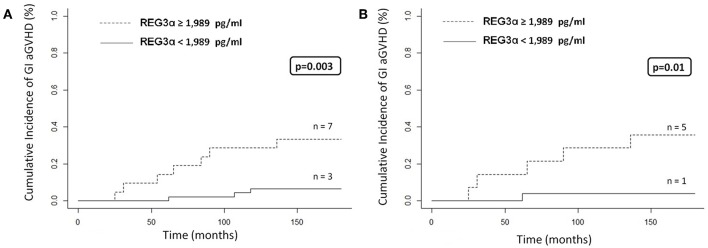
**(A)** Cumulative incidence of gastrointestinal GVHD according to REG3α levels at day +30 (cut-off 1,989 pg/ml) in the whole cohort (*n* = 10). **(B)** Cumulative incidence of gastrointestinal aGVHD according to REG3α levels at day +30 (cut-off 1,989 pg/ml) in RIC patients (*n* = 6).

Patients with grade II-IV aGVHD presented higher levels of REG3α at day +30 (1,358 vs. 500 pg/ml; *p* = 0.09). Interestingly, these levels were also higher in patients who developed gastrointestinal aGVHD than in patients who did not (2,483 vs. 1,011 pg/ml; *p* = 0.19). This trend was not observed in GVHD affecting other tissues (skin or liver). Similarly, patients with NRM and patients who died for any other reason presented higher levels of REG3α on day +30, with no significant differences [1,161 vs. 500 pg/ml (*p* = 0.1) and 1,183 vs. 702 pg/ml (*p* = 0.08), respectively]. ROC curve analysis revealed that the best cut-off value for REG3α levels at day +30 for gastrointestinal aGVHD was 1,989 pg/ml. Patients with levels higher than 1,989 pg/ml at day +30 presented a significantly higher incidence of gastrointestinal aGVHD in the whole cohort (HR, 8.37; *p* = 0.003; Figure 4A) and in the RIC cohort (HR, 6.59; *p* = 0.01; Figure 4B). No other clinical variables were associated with gastrointestinal aGVHD.

**Figure 5:**
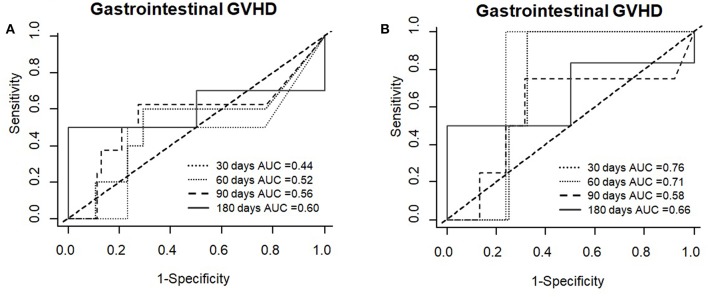
Time-dependent ROC curves for gastrointestinal GVHD using REG3α levels at day +30 in both, the whole cohort **(A)** and the RIC cohort **(B)**.

In testing associations of REG3α levels at day +30 with gastrointestinal aGVHD development after day 30, time-dependent ROC curves showed high AUC values in the RIC cohort (Figure 5).

**Figure 6:**
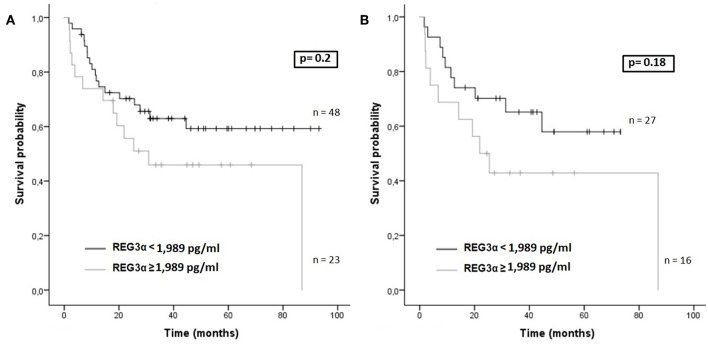
**(A)** Overall survival according to REG3α levels at day +30 (cut-off 1,989 pg/ml) in the whole cohort (*n* = 71). **(B)** Overall survival according to REG3α levels at day +30 (cut-off 1,989 pg/ml) in RIC patients (*n* = 43).

Finally, patients with lower levels of REG3α at day +30 presented better OS both in the whole cohort and in the RIC cohort, although the differences were not statistically significant (Figure 6).

## Discussion

Interest in plasma biomarkers for prediction, diagnosis, and prognosis of post-transplant complications has grown in recent years. Non-invasive biomarkers in peripheral blood can anticipate life-threatening complications and therefore improve therapeutic strategies in advance. Despite their proven usefulness, they are not yet part of the routine clinical practice. Most studies in this regard have been performed on HLA-identical or umbilical cord allo-HSCT only one study analyzed haplo-HSCT with PTCy (15). In the present study, we explored plasma levels of ST2 and REG3α in the largest single-center cohort of haplo-HSCT with PTCy investigated to date.

This is the first report in which biomarkers (ST2 and REG3α) are measured at day +15. Although other authors have postulated its possible utility, our results do not support it.

ST2 is a specific cellular marker that differentiates Th2 from Th1 cells; its soluble form is secreted by endothelial cells, epithelial cells, and fibroblasts. Soluble ST2 acts by promoting the Th1 phenotype, which has been associated with the pathophysiology of acute GVHD (26).

Our results show that ST2 levels at day +30 were higher in patients who presented acute GVHD II-IV, especially those who had received a RIC regimen. These patients tend to be older, present more comorbidities, and/or had received more previous lines of treatment. This may explain the greater endothelial damage observed and, therefore, the higher incidence of GVHD. The association of ST2 and occurrence of aGVHD is intriguing. Killer Ig-like receptors (KIR) play a central role in modulating NK effector function after haplo-HSTC. Acute GVHD is associated with the secretion of IL-12 and IL-18, which are known to promote NK cell functional maturation. This raises the possibility of effect on NK cells as IL-33/ST2 axis augments NK cell production of IFN-γ in response to IL-12 (27). Some studies did not find a statistically significant association between these entities (23, 25), whereas others, consistent with our study, observed a correlation between ST2 levels and GVHD (16, 24, 28, 29). However, such studies were not fully comparable with ours, since none of them included patients with haplo-HSCT or used PTCy as GVHD prophylaxis. In most of them, donors were HLA-identical (related and unrelated) and a minority were unrelated donors. One of the studies was performed on umbilical cord blood allo-HSCT (24).

The results of the only study performed on haplo-HSCT (15) differed from those of the present study. Unlike ours, the authors included a high proportion of patients with bone marrow as stem cell source, therefore, the number of patients presenting with aGVHD in their cohort was lower (*n* = 10), of which half started the clinic before day +30.

Furthermore, our results showed that high ST2 levels at day +30 were correlated with increased risk of NRM. In this case, our results are comparable to those obtained by the Baltimore group in the haplo-HSCT cohort, but also in HLA identical HSCT group with PTCy as GVHD prophylaxis (15). Other studies (23–25) performed in different transplant platforms showed the same results, therefore ST2 at day +30 seems to be a relevant biomaker for the development of NRM, regardless the stem cell source or GVHD prophylaxis.

The association between ST2 and aGVHD does not justify all causes of NRM in these patients. Kanakry et al. described 10 cases of NRM in a cohort of patients who underwent T cell–replete bone marrow haplo-HSCT. Most patients died of multi-organ failure and infection, and none of them died from GVHD. In this sense, differences in the causes of NRM may result from immune dysregulation or endothelial damage, in which ST2 is directly involved. Prospective studies are required to clarify the involvement of ST2 in the pathophysiology of post-transplant complications.

REG proteins act by protecting intestinal stem cells through binding of bacterial peptidoglycans. These proteins are thought to regulate uncontrolled inflammation by reducing pro-inflammatory cytokine production in ulcerative colitis (30). The correlation between mucosal damage and high REG3α levels suggests that microscopic breaches in the mucosal epithelial barrier caused by severe GVHD enable REG3α to cross into the bloodstream (21). In our study, we found that plasma REG3α levels at day +30 were higher in patients who developed gastrointestinal aGVHD than in patients without this complication. In our cohort, all patients but one presented gastrointestinal GVHD after day +30; therefore, measuring levels of this biomarker could help us to anticipate this complication and optimize immunosuppressive treatment. We also found elevated levels in patients with grade II-IV aGVHD (difference not statistically significant). Consistent with our results, Nomura et al. (28) reported higher levels of REG3α on day 14 in patients who developed aGVHD (affected organ not detailed). Despite these results, few studies have analyzed the predictive role of this biomarker, since most focus on its diagnostic role. High levels of REG3α have been reported at the onset of diarrhea, with a difference between lower gastrointestinal GVHD and non-GVHD diarrhea. In addition, higher concentrations were correlated with histological severity, poorer response to treatment and, therefore, greater 1-year NRM (19, 20). Since most of the patients in these studies had matched donors, it would be of great interest to perform the same analyses at diagnosis in a cohort treated with haplo-HSCT and PTCy in order to decrease the use of other invasive diagnostic techniques such as colonoscopy and biopsy. In our study, REG3α plasma levels were not associated with NRM (levels of REG3α on day +30 were higher in patients with NRM, although the differences were not significant). Kanakry et al. (15) found that REG3α plasma levels were significantly associated with a greater cumulative incidence of NRM in the HLA-haploidentical cohort. However, we found no such association. This association between high levels of REG3α and NRM but not with GVHD has also been described in studies with mostly HLA-identical donors (22, 26). More prospective and multicenter studies are required in patients receiving haplo-HSCT with PTCy.

It is also important to standardize the ELISA technique in plasma biomarkers to be able to establish a reproducible cut-off. Several cut-offs have been defined for ST2 (e.g., 33.9 ng/ml or 740 pg/ml) (23, 24). In contrast with previous findings, we found that the cut-off for NRM was 3,230 ng/ml. Cut-off levels of ST2 could also be affected by HLA disparity, stem cell source, and the intensity of the conditioning regimen. Other issues to consider include the commercial ELISA kits used, the sample (plasma or serum), and the processing of the sample (fresh or frozen).

Similar results were obtained with REG3α values. Ferrara et al. reported 151 ng/ml to be the cut-off for the diagnosis of gastrointestinal GVHD at onset of diarrhea, whereas in our study the best cut-off point on day +30 was 1,989 pg/ml. In contrast to our approach, these values were usually obtained at the onset of gastrointestinal symptoms (they behave as diagnostic values), while in our study the values were obtained on day 30 and demonstrated a predictive role. Therefore, algorithms to assign specific thresholds for intervention will need to be established, ideally in prospective multicenter trials.

Our analysis is subject to a number of limitations. We collected plasma samples on days +15 and +30 after transplant. Samples that are more appropriate for the clinical outcomes assessed should be collected more frequently early after transplant and in the following months. Such an approach could prove crucial for future proteomic biomarker studies. Consequently, the use of biomarkers in routine clinical practice should be validated in a larger cohort in a prospective multicenter study. Another limitation of this study is that the number of transplants is quite low. In order to confirm our results several statistical analyses have been performed. We have included the Fine-Gray model to directly estimate the effect ST2 on the cumulative incidence function of the outcome (in the presence of competing risks) and also attempt a time-dependent ROC curve methodology for competing risks to quantify potential predictive accuracy of ST2 and REG3α. Despite being the longest haplo-HSCT cohort studied so far, a validation in an independent cohort could be needed to verify the results.

Recent years have seen significant developments in the field of biomarkers for the prediction of post-HSCT outcomes. However, inconsistent results from clinical centers and studies have been reported, possibly resulting from heterogeneity in patient groups, underlying diseases, conditioning regimens, and GVHD prophylaxis, as well as from a lack of automation in laboratories using ELISA kits for proteomic biomarker analysis. According to our results, detection of ST2 and REG3α in plasma on day +30 after haplo-HSCT with PTCy can predict NRM and gastrointestinal aGVHD, respectively. Adding these biomarkers to risk algorithms could help to better classify groups of high-risk patients and thus modify risk with more intensive monitoring, immunosuppressive treatment, or other novel interventions. Moreover, the optimal cut-offs for high-risk, the standardization of laboratory methods, and the timepoints for analysis of plasma samples need to be better defined before these results can be applied in clinical practice.

## Data Availability Statement

All datasets generated for this study are included in the manuscript/supplementary files.

## Ethics Statement

The studies involving human participants were reviewed and approved by CEIm (Comité Ético de Investigación con medicamentos) of H.G.U. Gregorio Marañón. The patients/participants provided their written informed consent to participate in this study.

## Author Contributions

LS, CM-L, and IB: conception, design, and manuscript drafting. ND, PB, DS, and JA: data collection. LS, MK, DC, MC-L, JG, JD-M, CM-L, and IB: data analysis. LS, MK, DC, ND, PB, DS, MC-L, JA, JG, JD-M, CM-L, and IB: critical manuscript revision. All authors: final approval of the manuscript.

### Conflict of Interest

The authors declare that the research was conducted in the absence of any commercial or financial relationships that could be construed as a potential conflict of interest.
